# Slice Encoding for Metal Artefact Correction in magnetic resonance imaging examinations for radiotherapy planning

**DOI:** 10.1016/j.radonc.2016.05.004

**Published:** 2016-08

**Authors:** Maria A. Schmidt, Rafal Panek, Ruth Colgan, Julie Hughes, Aslam Sohaib, Frank Saran, Julia Murray, Jason Bernard, Patrick Revell, Mathias Nittka, Martin O. Leach, Vibeke N. Hansen

**Affiliations:** a The Institute of Cancer Research and The Royal Marsden NHS Foundation Trust, CR-UK & EPSRC Cancer Imaging Centre, Sutton, United Kingdom; b Royal Marsden NHS Foundation Trust, Radiotherapy Department, Sutton, United Kingdom; c Royal Marsden NHS Foundation Trust, Radiology Department, Sutton, United Kingdom; d Royal Marsden NHS Foundation Trust, Neuro-Oncology Unit, Sutton, United Kingdom; e Institute of Cancer Research and Royal Marsden NHS Foundation Trust, Radiotherapy and Imaging Department, Sutton, United Kingdom; f St George’s Hospital NHS Trust, Orthopaedic Surgery, London, United Kingdom; g Siemens Healthcare GmbH, Diagnostic Imaging, Camberley, United Kingdom; h Siemens Healthcare Limited, Diagnostic Imaging, Erlangen, Germany

**Keywords:** Image fusion for radiotherapy planning, MRI, Metallic implants in radiotherapy

## Abstract

**Background and purpose:**

Magnetic resonance (MR) and computed tomography (CT) images are degraded in the presence of metallic implants. We investigate whether SEMAC (Slice Encoding for Metal Artifact Correction) MR is advantageous for radiotherapy (RT) planning.

**Methods:**

Conventional and SEMAC MR protocols were compared (1.5 T). A spine fixation device suspended in gelatine, two patients with spine fixation devices and six patients with bilateral hip replacements were scanned with both conventional and SEMAC protocols. In spine patients the visibility of the spinal canal and spinal cord was assessed; in prostate patients, the visibility of the prostate, pelvic structures and the pelvic girdle.

**Results:**

The signal loss volume surrounding the spine fixation device was reduced by approximately 20% when the SEMAC protocol was employed, and registration errors were reduced. For spine patients, the spinal canal was completely visible only using the SEMAC protocol. In hip replacement patients, metal artifacts were local; the signal loss extended to the internal surface of the acetabulum in eight implants with conventional protocols, but only in four using SEMAC.

**Conclusions:**

SEMAC MR contributes towards correct co-registration of MR and CT images for RT planning, and is particularly relevant when the TV or OARs are close to implants.

The fusion of magnetic resonance (MR) and computed tomography (CT) images is often used to plan radiotherapy (RT), adding the superior MRI soft-tissue contrast used to outline target volumes (TV) and organs at risk (OAR) to the CT-based electronic density. Orthopaedic implants, relatively common in an ageing population, are often used in oncology patients to provide mechanical stability to the site of bone lesions or metastasis. Many commonly used metallic orthopaedic implants are MRI-safe but cause local artifacts in MR images due to susceptibility-related magnetic field inhomogeneity and to currents induced in the implant by radiofrequency fields and the imaging gradient fields [1]. Standard CT images of metallic implants are also degraded by streaks due to beam hardening and photon starvation artifacts, although advanced reconstruction techniques can minimise this problem [2], [3], [4]. The presence of metallic implants can be a serious challenge for obtaining high-quality anatomical images, a requirement for high precision conformal techniques such as Intensity Modulated RT (IMRT) and Volumetric Modulated Arc Therapy (VMAT). In addition, metallic implants also pose a challenge for RT, as the metal is very attenuating, limiting the range of beam angles available for RT delivery.

The susceptibility-related magnetic field inhomogeneity associated with orthopaedic implants is known to disrupt the spatial encoding of MR signals, leading to displacement of the spatial origin of signals along the readout gradient direction and to distortion of the selected slice (or slab) [5]. The in-plane distortion is characterised by bright areas (described as signal “pile up”) and dark areas of signal loss. Several strategies can be used to reduce distortion in conventional MRI techniques: spin-echoes are preferred over gradient-echoes, with short echo-times (TE) and high receiver and excitation bandwidth. However, the field inhomogeneity associated with common orthopaedic implants has been shown to be orders of magnitude higher than the naturally occurring field inhomogeneity associated with distributions of magnetic susceptibility values in biological material [6], and as a result conventional MRI techniques cannot eliminate artifacts completely.

In the last few years, specialist techniques have been developed to minimise metal artifacts in 2D and 3D MRI [7], [8], [9], employing high receiver and excitation bandwidth and encoding the field inhomogeneity. The resulting images do minimise susceptibility-related signal loss, but often at a cost of increasing the total acquisition time, noise levels and sometimes introducing blurring [10]. Slice Encoding for Metal Artifact Correction (SEMAC) is a multi-slice technique which resolves the susceptibility-related distortions both in-plane (readout direction) and through-plane (slice selection direction) [11]. In this work, we applied the SEMAC approach to the protocols we use for planning spine IMRT/VMAT and prostate RT. We tested these protocols in a test object designed for this purpose, and compared them to standard protocols on test objects and clinical examinations of patients with orthopaedic implants. Our objective was to determine whether the SEMAC approach can extend the use of MR to RT planning in the vicinity of metallic implants.

## Methods

### MRI pulse sequences for RT planning

All MR imaging for RT planning was undertaken on a 70 cm bore system adapted with a home-built perspex flat bed (1.5 T MAGNETOM Aera, Siemens Healthcare, Erlangen, Germany), employing transaxial T_1_ and T_2_ weighted fast spin-echo (FSE) pulse sequences. The development of IMRT and VMAT has enabled treatment of spinal and para-spinal masses [12], [13]; T_2_-weighted (T_2_W) FSE provides good delineation of the spinal cord (an OAR) and post-contrast T_1_-weighted (T_1_W) FSE sequences provide good lesion contrast for outlining the planning target volume (PTV). The use of T_2_W FSE is also well established in prostate RT planning [14], [15], [16], providing more detailed anatomical information than do the CT images.

In this study, a prototype SEMAC turbo spin-echo pulse sequence was implemented to match approximately the coverage, image quality and contrast of the conventional MRI protocol (WARP works-in-progress software package, Siemens Healthcare, Erlangen, Germany) [11]. SEMAC introduces high bandwidth excitation, view-angle tilting [10] to address in-plane distortions along the readout direction, and encodes the slice distortion with additional slice-encoding steps, thus enabling further correction. Initial experiments on test objects suggested that a number of 4 to 6 SEMAC slice-encoding steps [11] produced significant reduction in signal loss, in agreement with other reported studies [17]. However, we found necessary to reduce the number of averages and to increase the echo-train length in order to keep data acquisition within 5–6 min [18]. The reduction in averages is compensated by the gain in signal due to the SEMAC encoding, keeping the signal-to-noise ratio (SNR) of the image at an acceptable level. The in-plane voxel size was reduced to approximately 70% to counteract the increased blurring due to the introduction of view-angle tilting [10] and longer echo-trains. Basic protocol characteristics are presented in Table 1; protocol variations are mentioned in the figure captions.

### Test objects

A spine fixation device (pedicle screw and rod) was suspended in porcine gelatine inside a plastic container to evaluate SEMAC FSE sequences (Supplementary material, Fig. 1S). This implant contained metal screws and rods in three orthogonal directions (approximately superior/inferior, left/right and anterior/posterior) and thus reproduced realistically a difficult clinical examination (signal loss and image distortion). This object enabled assessment of metal artifacts against a uniform background, and was scanned transaxially with the T_1_W protocols of Table 1 (with and without metal artifact reduction). CT images were also obtained (Brilliance CT BigBore, Philips Healthcare, Netherlands). Threshold-based image segmentation was used to measure the implant volume in CT images, and the signal loss volume surrounding the implant in MR images.

MR and CT test object images were co-registered using a clinical RT treatment planning system (Pinnacle^3^ 9.8, Philips). A gold-standard CT-MR co-registration was defined as the position where the outline of the porcine gelatine volume inside the plastic container coincides in MR and CT images. In order to investigate how much the image artifacts interfere with the registration process, the MRI volume to be registered with CT was restricted to a central portion of the test object, containing the implant but excluding the outline of the volume defined by the gelatine medium. In this situation the shape of the container does not contribute towards the registration process, which depends only on image features (either real or artifactual ones). The gold-standard MR-CT co-registration was used as the starting position and a second automated registration of both conventional FSE and SEMAC FSE images with CT was undertaken. The final co-registration coordinates (translation *x*, *y* and *z* and rotation around the three main axes) were compared with the gold-standard co-registration. Manual registration was not used due to the difficulty in blinding the users to the container position.

### Clinical examinations

This work was approved by the Research Ethics Committee. Two patients with spinal metallic fixation devices and six patients with bilateral hip replacements were scanned with both conventional FSE and SEMAC FSE protocols; resulting images were compared. For the spine fixation devices, the visibility of the spinal canal and spinal cord were assessed for each slice, and the shortest distance between the metallic device and the spinal canal was measured in CT examinations. For the hip replacement patients, the prostate, pelvic structures and the internal surface of the pelvic bones were scrutinised in all slices, with particular attention to the medial surface of the acetabulum, closest to the implants. Each side of the pelvis was considered separately for the latter, as hip replacements are not necessarily identical on either side. The shortest distance from the metallic implant to the internal surface of the pelvic bones was measured manually for each implant, using the CT examination.

Unlike the co-registration of CT and MR images of the test object, there is no gold-standard “correct” co-registration for patient studies. The final co-registration used in clinical examinations was that performed by experienced RT physicists and radiation oncologists using all the information available. In this situation we simply ascertain whether the bony anatomy is visible on the examinations with the reasoning that the visibility of high-contrast structures provides an indication of the reliability of the MR-CT co-registration.

## Results

**Fig. 1:**
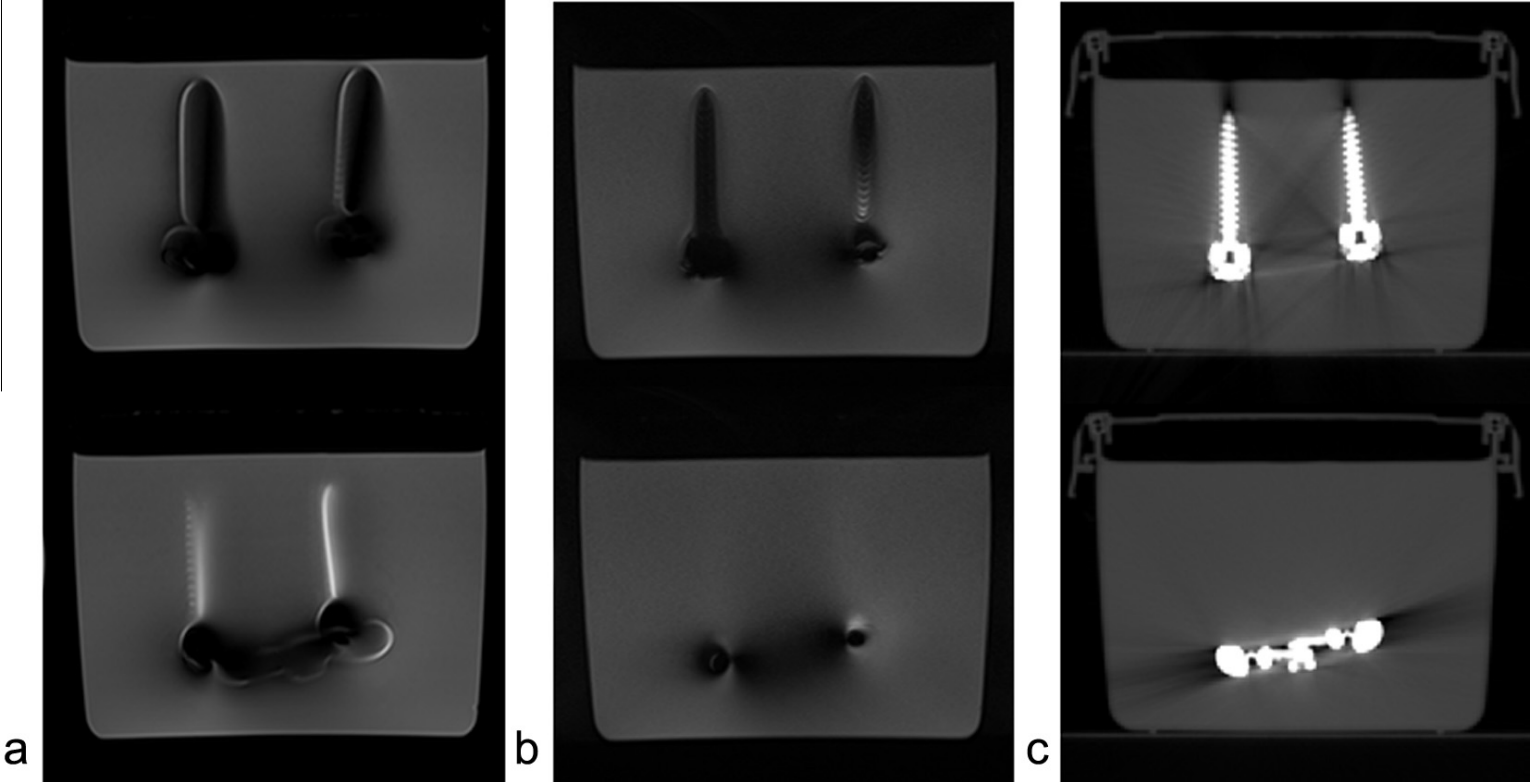
Conventional FSE (a) and SEMAC FSE (b) images for two separate slices (top and bottom). Signal loss and signal pile up are both greatly reduced with the introduction of SEMAC, with some residual effects in the vicinity of the implant. CT images (2.5 mm slice thickness) at approximately the same location are shown for comparison (c).

For the test object, the conventional FSE images presented evidence of distortion: extensive areas of signal loss and signal pile up. These were reduced when the SEMAC FSE protocol was used (Fig. 1). The implant volume was 34.5 ± 0.2 cm^3^ and the signal void around the implant was reduced by approximately 20% from 16.0 ± 0.5 cm^3^ (conventional FSE protocol) to 12.9 ± 0.5 cm^3^ (SEMAC FSE protocol). Segmentation is affected by artifacts in CT and MR, and cannot be fully automated. From the uncertainty in the threshold determination, the error in volumes can be estimated as 0.5 cm^3^ and 0.2 cm^3^ for MR and CT images, respectively.

**Fig. 2:**
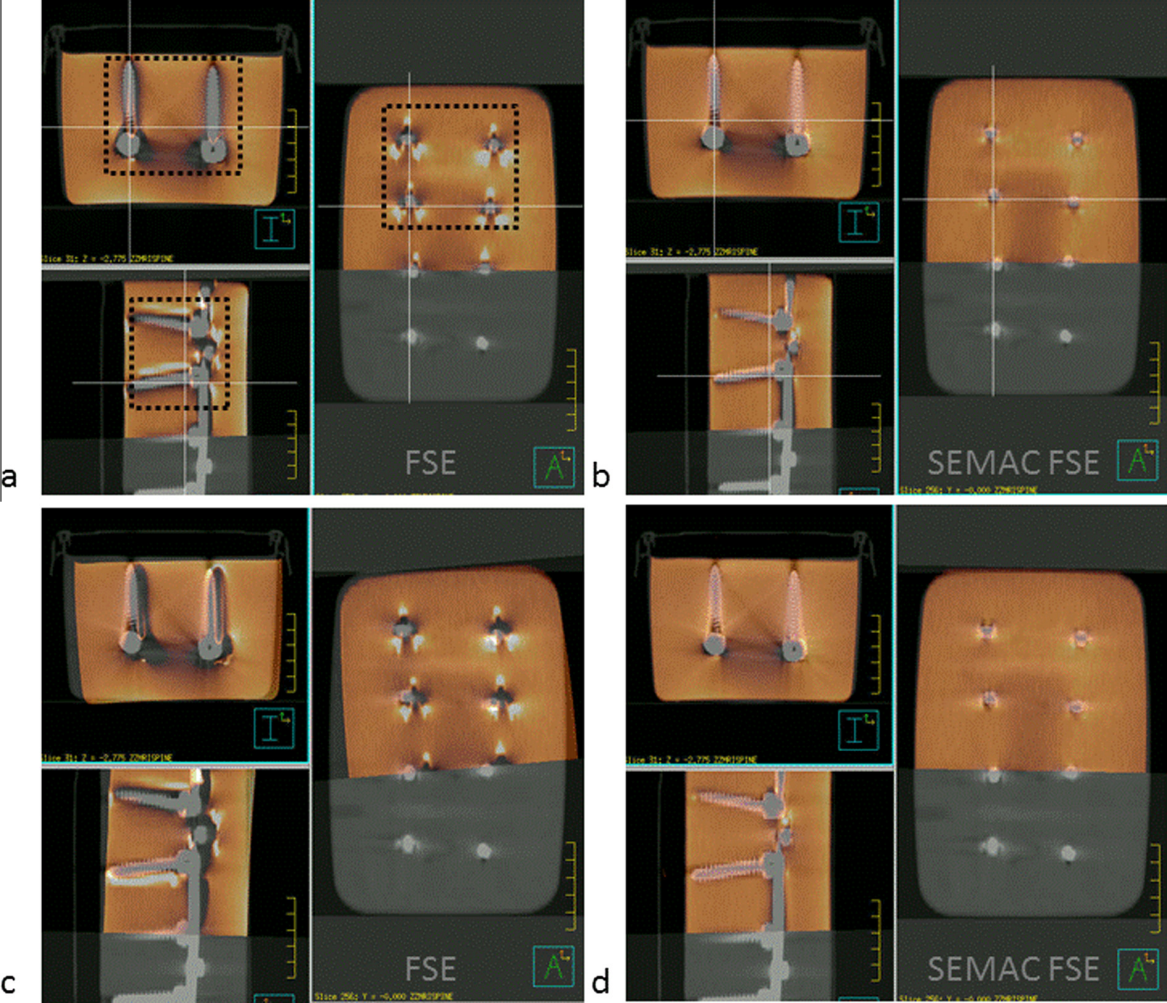
Conventional MR FSE (left) and MR SEMAC FSE images (right), overlaid on CT. The MR images cover a smaller volume. (a) and (b) show the best possible co-registration (gold standard), which uses the position of the gel-filled plastic box as a reference; (c) and (d) show the best automatic co-registration (Pinnacle, Philips), which is clearly disturbed by structured artifacts.

The plastic box containing the object is visible in CT but not in MRI. For the gold-standard co-registration of MR and CT the plastic box formed an outline around the gel test object (Fig.2a and b). In Fig.2c and d, the automated co-registration of the MR and CT datasets obtained by restricting the MR volume to the central part of the test object indicated in 2a is shown. The best automated co-registration was achieved with normalised cross-correlation as a cost function. This co-registration is approximately correct for the SEMAC FSE protocol, but completely incorrect for the conventional FSE protocol, suggesting that image artifacts significantly disturb the registration process. For the conventional FSE dataset, the automated co-registration algorithm aligns CT and MR artifacts, introducing error. In addition to translation, a rotation of 5° around the y direction (anterior/posterior) is clearly visible (Table 2).

**Fig. 3:**
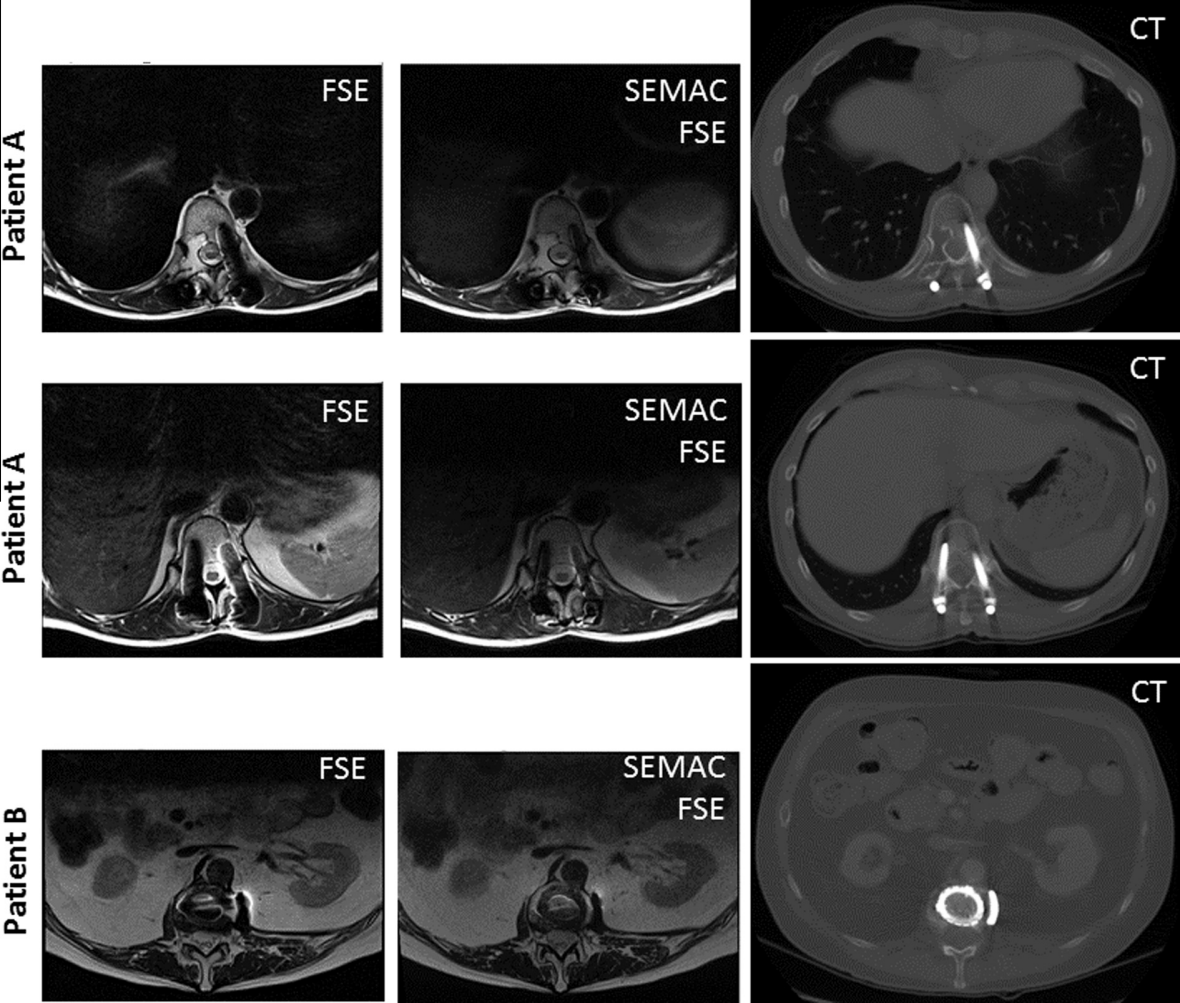
Spine images where the spinal canal was at least partially obscured by artifacts with FSE techniques: two slices on patient A and one on patient B. Areas of signal loss and signal pile up are visible with conventional FSE techniques, but minimised with SEMAC FSE techniques, enabling better registration between MRI and CT and complete visualisation of the spinal cord within the spinal canal.

In both patients with spinal fixation devices, the metallic implant was found to be adjacent to the spine canal. Fig. 3 shows slices where the spinal canal is partially affected by signal loss in conventional FSE protocols (two slices for patient A, and one for patient B). Using the SEMAC FSE protocol, the spinal canal is visible throughout the scanned volume. Areas of signal loss and signal pile up are reduced by using SEMAC. Experienced RT planning physicists registered MR and CT datasets manually by considering the position of the spinal canal in MRI and CT. On the registered MR-CT dataset, the signal loss extends to approximately 5 mm from the implant surface using conventional FSE and up to 3 mm using SEMAC FSE.

**Fig. 4:**
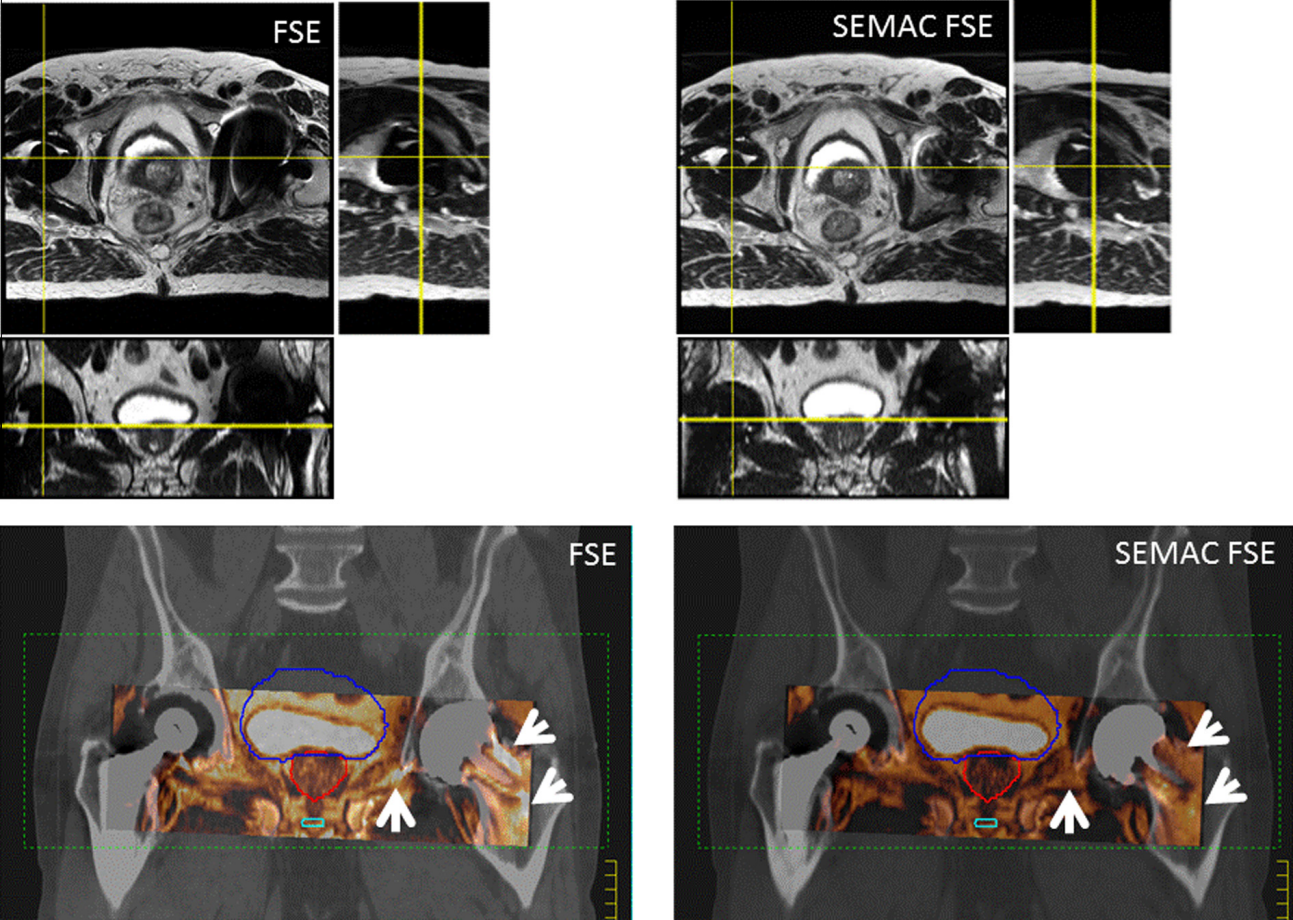
Conventional FSE (left) and SEMAC FSE (right) in patient with bi-lateral hip replacement (slice thickness 2.5 mm). The right hip (left in figure) is the least affected by signal loss, but geometrical distortion is still clearly visible in the conventional FSE, sagittal reconstruction. Patient position changed slightly between conventional FSE and SEMAC FSE acquisitions (bladder filling and rectal position). The registration with CT was undertaken by experienced personnel using all the information available. This task is hindered by the areas of signal pile up in conventional FSE images (arrows). Signal loss and signal pile up are reduced in SEMAC FSE images.

A total of 12 hip replacements were scanned in six patients, demonstrating geometrical distortion and signal loss, sometimes extensive. However, the metal artifacts did not reach the volume of interest: prostate, bladder, rectum and the seminal vesicles were clearly visible for all patients. Manual MR-CT co-registration was performed. The median distance between the internal surface of the pelvic girdle and the metallic surface was 7 mm (median = 7.6, interquartile range 4–10 mm), with distances ranging from zero (adjacent) to 21 mm. In 8 of those hip replacements, the signal loss extended to the internal surface of the acetabulum with conventional FSE protocols and therefore it was impossible to visualise the entire surface of the pelvic girdle. Using SEMAC FSE techniques the signal loss was reduced and in only four hip replacements it was not possible to visualise the complete internal surface of the pelvic bones. These tended to be situated closer to the internal surface of the acetabulum and included an examination where the implant reached the inner cortex of the acetabulum. In that examination, the acetabulum surface was not completely visible in any MR (or CT) images. Fig. 4 shows conventional FSE and SEMAC FSE registered with CT for planning. Areas of signal loss and signal pile up are visible. Physiological changes took place between the two MRI acquisitions, and the rectal distension is different in FSE and SEMAC FSE images. The worst affected and least affected hip replacements are presented in Fig. 2S (Supplementary material).

## Discussion

This work presents significant evidence of distortion in conventional FSE MRI around spine fixation devices and hip replacements; signal loss and signal pile up were present in all FSE examinations (test object and clinical). Signal loss, signal pile up and distortion were reduced when the SEMAC FSE protocols were introduced, but not completely eliminated. Our work is in agreement with previous reports: Månsson et al. employed SEMAC factors up to 18 in fairly long data acquisitions (up to 18 min) and some metal artifacts still remain [17], while Sutter et al. employed SEMAC factor 12 [19] and Reichter et al. SEMAC factor up to 15 [20] to similar results. In contrast, our work investigated practical FSE SEMAC protocols, suited to RT planning examinations: the data acquisition was limited to 6 min, with SEMAC factor 6, and the protocol was designed not to introduce additional blurring, as the ability to visualise OAR and TV in MR images could not be compromised. All other approaches to metal artifact reduction also involve some compromise between many competing requirements. At a basic level, imaging with higher gradients (i.e. increasing the readout and excitation bandwidth) will make susceptibility-related field inhomogeneity less relevant and will lead to some reduction on metal-induced artifacts, but at the price of a lower SNR and higher power deposition [6]. View-angle tilting (VAT [21]) on its own may reduce in-plane distortions but introduces some blurring, which can be reduced in longer acquisitions with multiple readouts [10]. Reports on metal artifact reduction techniques which attempt to encode and resolve through-plane displacement in 2D [7] and 3D [8], [9] present superior results, but the gradual reduction in signal loss and artifacts is associated with longer acquisitions, with some residual effects [6]. Therefore the trade-off between longer data acquisitions and the presence of metal-induced artefacts justifies our approach of considering practical SEMAC protocols which could be used clinically, in acquisitions of up to 6 min.

In common with other work presented in the literature [17], [19], [20] we found intrinsic difficulties in attempting to quantify signal loss in clinical images, against a background that includes areas of low image intensity (pelvic T_2_W examinations, for example). Furthermore, it is impossible to separate changes related to the introduction of the SEMAC technique from other changes: Fig. 4 shows a particular example where the position of the prostate is not the same between conventional FSE and SEMAC FSE examinations, due to physiological changes. Unlike Sutter et al. [19], we found manual outlining of the signal loss area excessively subjective, and could only rely on histogram-based analysis for test object images [17]. We reliably assessed the signal loss volume in three dimensions for the test object, against a uniform background, using the CT examination to evaluate the implant volume, and found that it is reduced by approximately 20% when FSE SEMAC is employed.

Metal artifact reduction techniques may contribute towards RT planning in two different ways: (i) by enabling the correct depiction of relevant structures for outlining (TVs and OARs), and (ii) by contributing towards CT-MR registration with a more accurate depiction of high contrast structures. The spine examination is an example of the former, and can be considered as a worst-case scenario. The purpose of the MRI examination is to enable outlining the spinal cord, an OAR, and spine lesions, and the metallic fixation devices are adjacent to the spinal canal. For those examinations, only the SEMAC FSE protocol enabled visualisation of the whole spinal canal. Therefore the estimated 20% reduction in the signal loss volume has a direct impact, enabling accurate localisation of the spinal cord (OAR) within the spinal canal. The treatment of those patients with highly conformal techniques (IMRT or VMAT) is very challenging, and the introduction of FSE SEMAC protocols makes those techniques viable for a larger number of patients. In contrast, in prostate examinations SEMAC FSE contributes towards CT-MR co-registration only, as the TVs and OARs are visible with both conventional and SEMAC FSE.

Considering that the signal loss around metallic implants is unlikely to be completely eliminated with our practical SEMAC protocols, the most pertinent question is whether the introduction of SEMAC improves our ability to co-register MR and CT datasets. The metallic implant as the only feature of the test object – the surrounding gel is otherwise unstructured. The MR-CT automated co-registration is very poor for the conventional FSE MR, but approximately correct after the introduction of SEMAC, demonstrating that when the metal artifacts were significantly reduced a better registration ensued. This can only be clearly demonstrated on a test object, as the implant is the only feature contributing towards CT-MR registration. The test object is in this sense the worst case scenario for CT-MR co-registration, which is excessively affected by CT and MR artifacts.

In a clinical setting, it is essential to ensure that there are enough visible undistorted structures in the MR images to enable correct co-registration of MR and CT datasets, with particular attention to bone which is a high-contrast structure in CT. Increased visibility of the spinal canal and pelvic girdle was demonstrated quantitatively in this study. In this discussion we consider that signal loss and geometrical distortion may affect the RT outcome by affecting CT-MR registration, even if they do not affect the TV and OARs.

In pelvic examinations, MRI enables better outlining of prostates, prostate lesions, seminal vesicles and urethra, which were not affected by the signal loss associated with the vicinity of metal. However the CT-MR registration process makes use of the internal surface of the pelvic girdle, which was more consistently visible using the SEMAC FSE technique. Incorrect registration may lead to a shift of the pelvic TV and OAR in pelvic examinations, leading to error. The margins used for prostate cancer from the clinical target volume (the whole prostate) to the planning target volume are 2–10 mm. Using Image Guided RT for delivery, the posterior margin may be reduced to 2 mm, to minimise the rectal toxicity. Therefore introducing displacements in the posterior/anterior direction is particularly worrying. This suggests that SEMAC FSE is also advantageous to allow more accurate MR-CT registration of pelvic MR examinations performed for RT planning. However, the use of SEMAC FSE should not be considered in isolation; other alternative strategies to improve confidence in the CT-MR registration can also be pursued. One of the alternative strategies is to enlarge the coverage of the examination in the superior/inferior direction to comprise implant-free bone structures. Both approaches (the use of FSE SEMAC and increasing the volume covered) can be combined; however, this would entail longer MRI examinations, which could be a limiting factor.

In this study, the discussion on the effect of the FSE SEMAC on the registration of clinical images takes place without a registration gold standard. Although we believe the SEMAC FSE protocol makes the MR-CT registration more reliable by improving the visualisation of relevant anatomical structures, other factors also affect the outcome. In particular the level of experience of RT personnel and their familiarity with MR metal artifacts is likely to play a role. RT physicists who participated in this project and scrutinised conventional FSE and SEMAC FSE images side by side gained an awareness of the nature of the distortion expected: (i) the signal loss is not symmetric in relation to the implant, (ii) the displacement of signals occurs in the readout direction, and (iii) the selected slice may be warped in the vicinity of the implant. Better understanding is certainly a contributing factor towards a more accurate registration, and the process of comparing images acquired with and without metal artifact reduction techniques side by side highlights the distortions, their origin and their location. In general we found that inexperienced personnel tended to line up CT and MR artifacts, and therefore introduced error to the registration process. Only a much larger study can address issues related to levels of experience required to perform a complex task such as the registration of distorted MR images with CT, also taking into account the CT artifacts. Metal artifacts were present in all conventional MRI examinations, and were still visible in many SEMAC FSE examinations. For this reason automated registration must be treated with caution.

In conclusion, this work shows improvement in geometrical accuracy and reduction of signal loss around common metallic implants using practical SEMAC FSE sequences. This contributes towards more accurate co-registration and fusion of MR and CT images for RT planning, and is particularly relevant when the TV or OARs are very close to metallic implants. For the latter, SEMAC FSE allows more accurate delineation of TVs and OARs.

## Conflict of interest statement

None.

## Figures and Tables

**Table 1:** Conventional FSE and SEMAC FSE protocols for RT planning.

	Conventional T_2_W FSE	SEMAC T_2_W FSE	Conventional T_1_W FSE	SEMAC T_1_W FSE
FOV (mm)	250 (spine) 300 (prostate)	250 (spine) 300 (prostate)	250 (spine) 300 (prostate)	250 (spine) 300 (prostate)
Acquisition matrix, (reconstruction matrix)	320 × 224 (320 × 320)	448 × 358 (448 × 448)	320 × 224 (320 × 320)	448 × 358 (448 × 448)
Slice thickness (mm)	3	3	3	3
TE (ms)	88	91	23	25
TR (ms)	5200	5200	600	600
Receiver bandwidth (Hz/pixel)	200	585	200	587
Echo-train length	16	32	4	32
Averages	3	1	4	1
Total acquisition time (min)	3:20	5:19	3:53	5:32
SEMAC slice encoding steps	n.a.	6	n.a.	6

**Table 2:** Difference in co-registration parameters between automated co-registration and the gold standard co-registration.

	Conventional FSE protocol	SEMAC FSE protocol
*X* translation (left/right)	−0.15 cm	0.05 cm
*Y* translation (anterior/posterior)	0.26 cm	−0.01 cm
*Z* translation (head/foot)	0.39 cm	0.11 cm
*X* rotation	2.55°	0.04°
*Y* rotation	−5.02°	0.83°
*Z* rotation	1.68°	−0.06°

## Appendix A Supplementary data

Supplementary Fig. 1S (a) Sagittal (left) and coronal (right) X-ray images of test object containing a spine fixation device suspended in gelatine within a plastic container. (b) Example of clinical X-ray (sagittal) of similar spine fixation device, in situ.

Supplementary Fig. 2S Conventional FSE and SEMAC FSE (slice thickness 2.5 mm) in two patients, both with bi-lateral hip replacement. Geometrical distortion in clearly visible in conventional FSE, sagittal view. (a) The left hip (right on picture) is the most affected, SEMAC FSE reduces signal loss, but does not allow complete visualisation of acetabulum. (b) The right hip (left on picture) is least affected by signal loss. However, geometric distortion is apparent on sagittal reconstruction and would hinder CT-MR registration.
