# Supplementary material for: Slice Encoding for Metal Artefact Correction in magnetic resonance imaging examinations for radiotherapy planning
Source: Radiother Oncol. 2016 Aug;120(2):356–62. doi: 10.1016/j.radonc.2016.05.004 (PMC5013753; doi:10.1016/j.radonc.2016.05.004)
Supplement: Supplementary Fig. 1S — (a) Sagittal (left) and coronal (right) X-ray images of test object containing a spine fixation device suspended in gelatine within a plastic container. (b) Example of clinical X-ray (sagittal) of similar spine fixation device, in situ. [file mmc1.pdf]

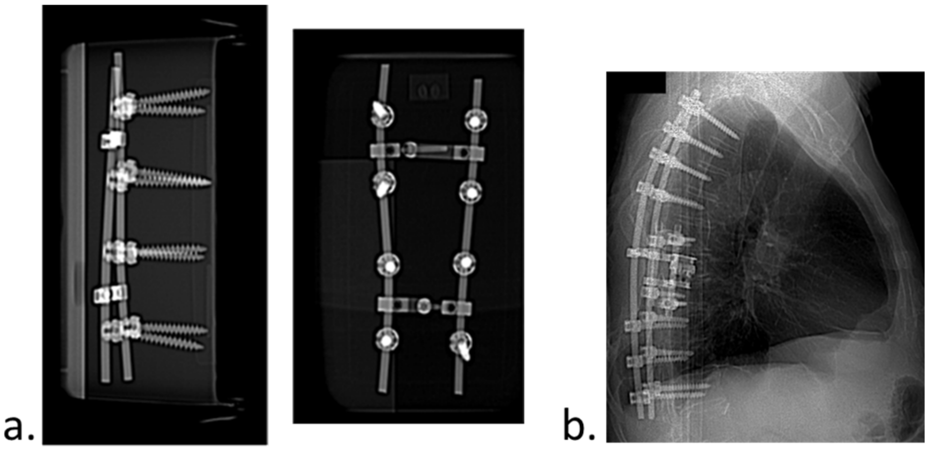

Figure 1S- a. Sagittal (left) and coronal (right) X-ray images of test object containing a spine fixation device suspended in gelatine within a plastic container. **b.** Example of clinical X-Ray (sagittal) of similar spine fixation device, in situ.
