# Supplementary material for: Slice Encoding for Metal Artefact Correction in magnetic resonance imaging examinations for radiotherapy planning
Source: Radiother Oncol. 2016 Aug;120(2):356–62. doi: 10.1016/j.radonc.2016.05.004 (PMC5013753; doi:10.1016/j.radonc.2016.05.004)
Supplement: Supplementary Fig. 2S — Conventional FSE and SEMAC FSE (slice thickness 2.5 mm) in two patients, both with bi-lateral hip replacement. Geometrical distortion in clearly visible in conventional FSE, sagittal view. (a) The left hip (right on picture) is the most affected, SEMAC FSE reduces signal loss, but does not allow complete visualisation of acetabulum. (b) The right hip (left on picture) is least affected by signal loss. However, geometric distortion is apparent on sagittal reconstruction and would hinder CT-MR registration. [file mmc2.pdf]

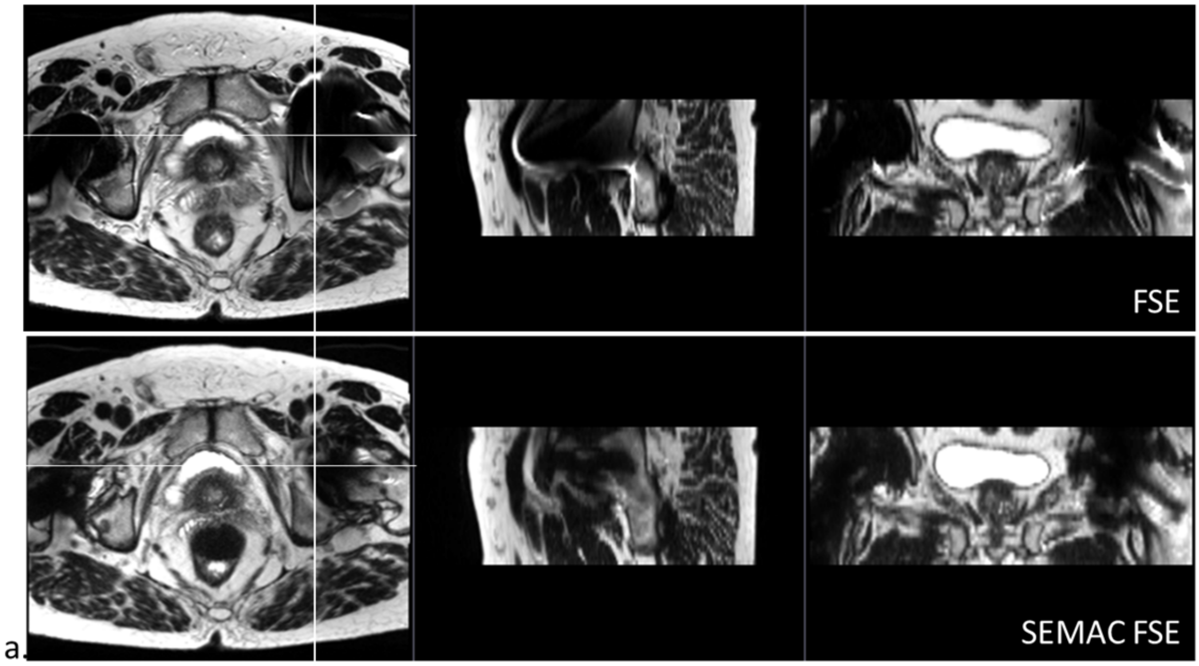

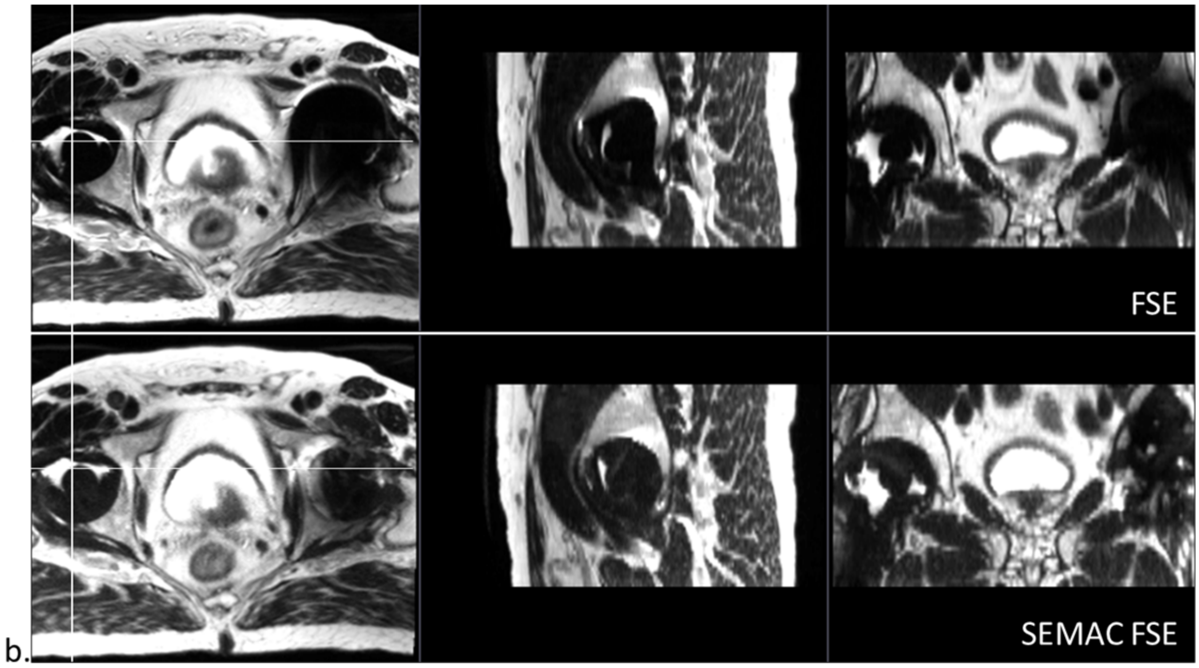

Figure 2S – Conventional FSE and SEMAC FSE (slice thickness 2.5 mm) in two patients, both with bi-lateral hip replacement. Geometrical distortion in clearly visible in conventional FSE, sagittal view. **a.** The left hip (right on picture) is the most affected, SEMAC FSE reduces signal loss, but does not allow complete visualisation of acetabulum. **b.** The right hip (left on picture) is least affected by signal loss. However, geometric distortion is apparent on sagittal reconstruction and would hinder CT-MR registration.
